# Strategies for senolytic drug discovery

**DOI:** 10.1111/acel.13948

**Published:** 2023-08-07

**Authors:** Helen Power, Peter Valtchev, Fariba Dehghani, Aaron Schindeler

**Affiliations:** ^1^ Faculty of Engineering, School of Chemical and Biomolecular Engineering The University of Sydney Sydney New South Wales Australia; ^2^ Centre for Advanced Food Engineering The University of Sydney Sydney New South Wales Australia; ^3^ Bioengineering and Molecular Medicine Laboratory The Children's Hospital at Westmead and The Westmead Institute for Medical Research Westmead New South Wales Australia

**Keywords:** cellular senescence, drug discovery, senescence, senolytics

## Abstract

Senolytics are a category of drugs that reduce the impact of cellular senescence, an effect associated with a range of chronic and age‐related diseases. Since the discovery of the first senolytics in 2015, the number of known senolytic agents has grown dramatically. This review discusses the broad categories of known senolytics—kinase inhibitors, Bcl‐2 family protein inhibitors, naturally occurring polyphenols, heat shock protein inhibitors, BET family protein inhibitors, P53 stabilizers, repurposed anti‐cancer drugs, cardiac steroids, PPAR‐alpha agonists, and antibiotics. The approaches used to screen for new senolytics are articulated including a range of methods to induce senescence, different target cell types, various senolytic assays, and markers. The choice of methods can greatly influence the outcomes of a screen, with high‐quality screens featuring robust systems, adequate controls, and extensive validation in alternate assays. Recent advances in single‐cell analysis and computational methods for senolytic identification are also discussed. There is significant potential for further drug discovery, but this will require additional research into drug targets and mechanisms of actions and their subsequent rigorous evaluation in pre‐clinical models and human trials.

## Graphical abstract

Senolytics are a category of drugs that reduce the impact of cellular senescence, an effect associated with a range of chronic and age‐related diseases. This review discusses current senolytics and screening approaches for identifying novel active compounds. Ultimately, senolytic activity can be cell‐type and assay‐dependent, requiring extensive validation. Further drug discovery requires ongoing research into drug targets and evaluation in pre‐clinical and clinical studies.

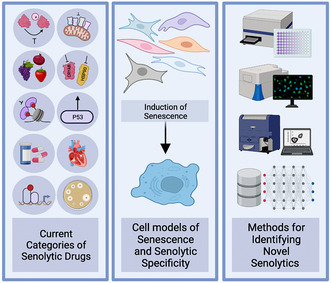

**Abbreviation**
25HC: 25‐hydroxycholesterol
ANT2: adenine nucleotide translocase‐2
BETd: bromodomain and extra‐terminal domain family protein degrader
C_12_FDG: 5‐dodecanoylamino‐fluoresceindi‐β‐D‐galactopyranoside
CRYAB: α‐crystallin β chain
D: dasatinib
HDFs: human dermal fibroblasts
HSPs: heat shock proteins
HUVEC: human umbilical vein endothelial cell
IVD: intervertebral disc
LC3: microtubule‐associated protein 1A/1B‐lightchain 3
MDM2: human homolog of mouse double minute 2
MEF: murine embryonic fibroblast
MiDAS: mitochondrial dysfunction‐associated senescence
MitoTam: mitochondria‐targeted tamoxifen
MSC: mesenchymal stem cell
MUG: 4‐methylumbelliferyl‐β‐d‐galactopyranoside
OIS: oncogene‐induced senescence
OXR1: oxidation resistance 1
PL: piperlongumine
PPARα: peroxisome proliferator‐activated receptorα
Q: quercetin
RIS: replication‐induced senescence
ROS: reactive oxygen species
SA‐β‐gal: senescence‐associated β‐galactosidase
SAHF: senescence‐associated heterochromatin foci
SASP: senescence‐associated secretory phenotype
SIS: stress‐induced senescence
USP7: ubiquitin‐specific protease 7
X‐gal: 5‐bromo‐4‐chloro‐3‐indoyl β‐D‐galactopyranoside

## 1 INTRODUCTION

Senescent cells are defined by a permanent cessation of cell division. These cells can exhibit features associated with the G1 and G2 growth phases of the cell cycle, distinguishing them from both quiescent and terminally differentiated cells (Mao et al., 2012). Replicative senescence appears to be an essential feature of somatic cells, but senescence can also be induced by the range of stimuli including oxidative stress (Chen & Ames, 1994), radiation (Chen et al., 2008; Day et al., 2014), chemotherapeutics (Guillon et al., 2019; Leontieva et al., 2010), mitochondrial dysfunction (Wiley et al., 2016), oncogene overexpression (Courtois‐Cox et al., 2008) and stimuli derived from existing senescent cells (Admasu et al., 2021).

The accumulation of senescent cells in mammalian tissues and organs strongly correlates with age‐related dysfunction. This is attributed to a senescence‐associated secretory phenotype (SASP), which is characterized by the expression of pro‐inflammatory cytokines, chemokines, growth factors, matrix metalloproteinases, bioactive lipids, and other factors that promote tissue dysfunction and chronic low‐level inflammation (Kirkland & Tchkonia, 2020). Changes in mitochondrial, lysosomal, and endoplasmic reticulum activity also occur (Hernandez‐Segura et al., 2018). Moreover, increased expression of negative regulators of apoptosis and anti‐apoptotic genes promotes the survival of senescent cells (Zhu et al., 2015). However, the changes that occur during senescence are complex, with a high degree of heterogeneity between cells and even within the same cell over time (Kirkland & Tchkonia, 2020).

Various reviews have explored the role of senescent cells in a range of chronic, inflammatory, and/or age‐related diseases. These include chronic kidney disease (Wang et al., 2021), diabetes (Murakami et al., 2022), age‐related osteoporosis (Pignolo et al., 2021), osteoarthritis (Xie et al., 2021), dementia (Behfar et al., 2022), cardiovascular disease (Dookun et al., 2022), chronic lung diseases (Barnes et al., 2019) and cancer (Wyld et al., 2020). Prophylaxis or treatment of these conditions represents a growing challenge with an aging world population. By 2050, the number of adults older than 60 is predicted to reach 2.1 billion, accounting for 22% of the global population (World Health Organization, 2021). Multiple studies have indicated that half of all individuals above 65 suffer from more than one chronic condition (Atella et al., 2019; Kingston et al., 2018; Picco et al., 2016; Salive, 2013), and this may be even higher in the US (Buttorff et al., 2017). These conditions critically impact “healthspan”—the period of life where one is healthy—which is increasingly considered to be a metric as important as lifespan.

Studies highlighting the benefits of eliminating harmful senescent cells (Baker et al., 2011), have led to many new initiatives in drug discovery and design. Senolytics are a class of drugs that selectively eliminate senescent cells and are promising candidates for the prevention and treatment of chronic conditions. In this review, we have provided an overview of small molecules with intrinsic senolytic activity, followed by methods for identifying and studying these compounds, with emphasis on the early stages of discovery and validation. We discuss the strengths and weaknesses of common approaches and explore recent advances in methodology. Although outside of the scope of this review, mechanisms of senotherapeutic compounds (Birch & Gil, 2020; He, Zheng, & Zhou, 2020; Zhang et al., 2022) and pathways for clinical translation have been discussed elsewhere (Kirkland & Tchkonia, 2020).

## 2 DISCOVERY OF THE FIRST SENOLYTIC DRUGS

The 2015 discovery of the first senolytic drugs, dasatinib (D) and quercetin (Q), arose from an effort to better understand senescent cell biology (Zhu et al., 2015). Using genome‐wide microarray analysis to compare transcriptomes of non‐senescent and irradiation‐induced senescent preadipocytes, Zhu et al. identified negative regulators of apoptosis and anti‐apoptotic factors to be among the most upregulated genes. Subsequently, the senolytic activities of D and Q were identified from a screen of 46 manually selected modulators of anti‐apoptotic pathways. Dasatinib, a tyrosine kinase inhibitor originally developed as an anti‐cancer drug, showed senolytic activity in preadipocytes, and to a lesser extent in human umbilical vein endothelial cells (HUVECs). Quercetin, a natural flavonoid and known inhibitor of serpins and various kinases including PI3K, exhibited senolytic activity in HUVEC cell assays. The distinct activity spectrums of these two compounds prompted their use in combination (D + Q), and this is frequently used to target a wider range of cells.

Shortly after this discovery, other screening efforts led to the identification of ABT‐263 (navitoclax) and piperlongumine (PL). Using WI‐38 fibroblasts induced to senescence by irradiation, ABT‐263 was discovered from a library of 37 individually chosen compounds related to senescent cell viability and with a broad range of bioactivities (Chang et al., 2016). The library included modulators of nutrient metabolism, inducers of reactive oxygen species (ROS) and inhibitors of anti‐apoptotic proteins, DNA damage response factors, and histone deacetylases. ABT‐263 is an inhibitor of several anti‐apoptotic proteins including Bcl‐2, Bcl‐w, and Bcl‐X_L_, which is consistent with the upregulation of anti‐apoptotic factors in senescent cells (Zhu et al., 2015). A study by the same authors identified PL and four analogs as possessing senolytic activity (Wang et al., 2016). PL originates from the long pepper plant and has been studied for its anti‐cancer effects and ability to modulate signaling pathways influencing cell‐cycle regulation, nutrient signaling, and the immune response (Parama et al., 2021). A follow‐up study investigating senolytic targets of PL, found it binds to and promotes degradation of oxidation resistance 1 (OXR1) and to induce apoptosis (Zhang et al., 2018).

In contrast to unbiased screening efforts, one of the first candidate approaches to identify novel senolytics focused on targeting the BCL‐2 family anti‐apoptotic factors Bcl‐w and Bcl‐X_L_ (Yosef et al., 2016). These factors are upregulated in senescent cells and can be inactivated by BH3‐only proteins, which promotes apoptosis. ABT‐737 was selected as a chemical mimetic of BH3 factors and is chemically similar to ABT‐263 (another inhibitor for Bcl‐2, Bcl‐w, and Bcl‐X_L_). ABT‐737 displayed senolytic activity in IMR‐90 fibroblasts induced to senescence by replicative exhaustion, etoposide treatment, and oncogenic H‐Ras expression.

## 3 CURRENT CATEGORIES OF SENOLYTIC DRUGS

**FIGURE 1:**
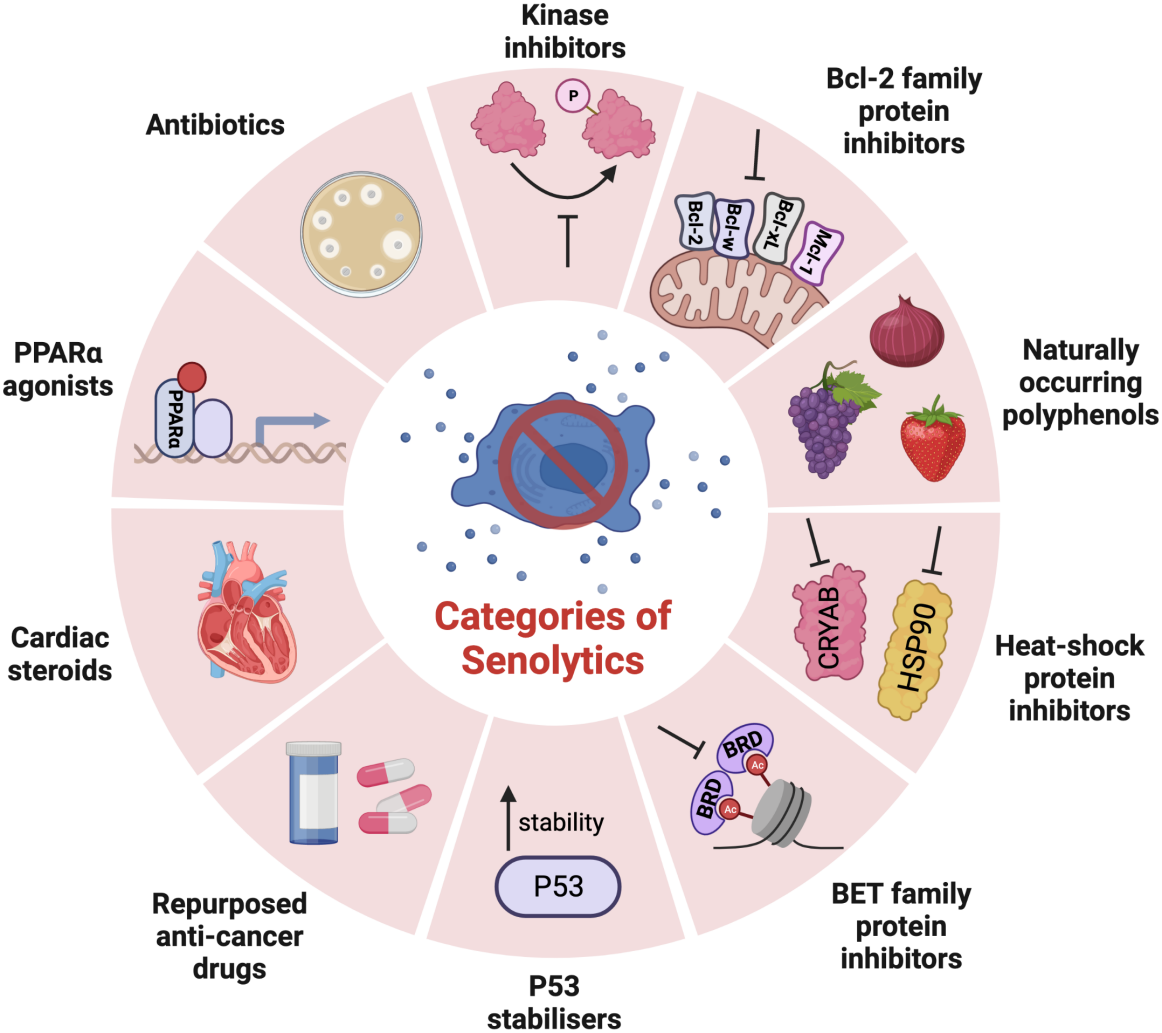
Current categories of senolytics.

Since the initial discovery of senolytics, therapeutic benefits of multiple senolytic compounds have been reported in pre‐clinical models, including lifespan extension (Yousefzadeh et al., 2018), increased bone mass and strength (Farr et al., 2017), improved liver function (Ogrodnik et al., 2017), enhanced kidney and heart regeneration post‐injury (Dookun et al., 2020; Mylonas et al., 2021), reduced toxicity, and relapse after chemotherapy (Demaria et al., 2017; Lerida‐Viso et al., 2022), improved metabolic function in diabetic mice (Pathak et al., 2018) and heightened cognitive ability in models of neurodegenerative disease (Bussian et al., 2018; Zhang et al., 2019). Further promising results have been published from the first human trials with D + Q, in which treatment reduced markers of senescence in blood, skin, and adipose tissue (Hickson et al., 2019) and improved physical function in a small cohort of patients with idiopathic pulmonary fibrosis (Justice et al., 2019). Despite these encouraging results, limitations associated with activity spectrum, negative side‐effects, bioavailability, and potency (Kovacovicova et al., 2018; Raffaele et al., 2021; Roberts et al., 2012; Sharma et al., 2020) necessitate ongoing research into novel senolytics and optimization of existing compounds. To this end, studies continue to discover new senolytic compounds beyond those currently known (as illustrated in Figure 1). The categories we have defined do not include complex molecular agents consisting of toxic compounds engineered to selectively target senescent cells. Such drugs are beyond the scope of this review, as they fundamentally differ in their mechanism and drug discovery pipelines. However, several studies have explored the potential of antibody conjugates (Poblocka et al., 2021), immunogen conjugates (Suda et al., 2021), proteosome targeting drugs (He, Zhang, et al., 2020), and galactose modified prodrugs (Cai et al., 2020; Guerrero et al., 2020).

### 3.1 Kinase inhibitors

Kinases regulate a broad range of cellular processes relevant to senescence including cell cycle arrest, pro‐inflammatory signaling, nutrient signaling, and the DNA damage response (Anerillas et al., 2020; Gaestel et al., 2009; Lu & Hunter, 2018). The early senolytic dasatinib, is an inhibitor of various Abl, Tec, and Src family kinases (Das et al., 2006; Hantschel et al., 2007; Tokarski et al., 2004). Additional senolytics were identified by screening the Selleck Chem L1200 kinase inhibitor library (Cho et al., 2020). From the screen, two compounds nintedanib and R406, were found to induce senolysis in senescent human dermal fibroblasts (HDFs). Nintedanib induced apoptosis via JAK2/STAT3 inhibition (Cho et al., 2022) and R406 induced apoptosis via focal adhesion kinase and p38 mitogen‐activated protein kinase inhibition (Cho et al., 2020).

Two additional screens investigating kinase inhibitors are also notable. From a library of 80 inhibitors, terreic acid, daidzein, PD‐98059, and Y‐27632 reduced the percentage of senescent cells and SASP expression in cells simultaneously exposed to stress stimuli (Kusumoto et al., 2021). Based on the model, it remains unclear whether the drugs are true senolytics or reduce the transition of cells to a senescent state. Another study which also used the Selleck Chem L1200 library found KU‐60019 attenuates senescence, but this may be associated with suppressing a senescent phenotype rather than acting as a true senolytic (Kang et al., 2017).

### 3.2 Bcl‐2 family protein inhibitors

Anti‐apoptotic members of the Bcl‐2 protein family: Bcl‐2, Bcl‐w, Bcl‐X_L_, and Mcl‐1, have established roles in maintaining senescent cell viability and are therefore promising senolytic targets (Troiani et al., 2022; Zhu et al., 2015). As noted, the Bcl‐2 family inhibitors ABT‐263 and ABT‐737 were previously shown to possess senolytic activity. However, two major clinical side effects of ABT‐263, thrombocytopenia and neutropenia, prompted a search for alternatives. Compared to ABT‐263, Bcl‐X_L_ inhibitors, A1331852 and A1155463 are still potent senolytics and may have a more favorable safety profile—though this needs to be validated clinically (Zhu et al., 2017).

Mcl‐1 inhibitors S63845, UM177, and AZD5991 possess senolytic activity in chemotherapy‐induced senescent pancreatic tumor cells, including cells resistant to senolysis by ABT‐263 (Troiani et al., 2022). The study found approximately 50% of tumor cells expressed *Bcl‐2* at low levels, whereas *Mcl‐1* was upregulated in most cells. Thus Mcl‐1 may be a superior target for reducing the impact of senescent cells arising from cancer treatment.

### 3.3 Naturally occurring polyphenols

Polyphenols are a diverse class of compounds present in fruits, vegetables, and other plants. They can produce a range of bioactive properties and their consumption has been associated with amelioration of many chronic diseases (Fraga et al., 2019). Quercetin, one of the first senolytics, belongs to this class of molecules. Subsequently, two independent studies revealed the senolytic activity of fisetin—a chemical analog of quercetin. One screened a group of 10 flavonoids (Yousefzadeh et al., 2018), whereas the other selected fisetin as a candidate (Zhu et al., 2017). Both quercetin and fisetin possess a similar activity spectrum, being effective in irradiated HUVECs, but not preadipocytes or IMR‐90 fibroblasts, suggestive of a common mechanism (Zhu et al., 2017).

This flavonoid screen also reported curcumin to have weak senolytic activity. Curcumin analogs with senolytic properties were since reported – EF24 (Li et al., 2019) and the metabolite o‐Vanillin (Cherif et al., 2019). These compounds have improved pharmacokinetic features and act via the promotion of the proteasomal degradation of anti‐apoptotic factors Bcl‐X_L_ and Mcl‐1. It is unclear whether the senolytic properties of curcumin underlie its ability to extend lifespan, such as in the fruit fly *Drosophila melanogaster* (Suckow & Suckow, 2006) and the roundworm *Caenorhabditis elegans* (Liao et al., 2011).

Due to the abundance of polyphenols in plants, one practical drug discovery approach involves screening plant extracts and subsequently analyzing active extracts in terms of their major components. By screening 46 natural compounds and plant extracts with putative anti‐aging properties, (Xu et al., 2021) identified grape extract and more specifically the polyphenol procyanidin C1 to have senolytic activity. A more recent study of four plant extracts with anti‐inflammatory properties reported the senolytic activity of the polyphenol gingerenone A, which is found in ginger (Moaddel et al., 2022). Gingerenone A is structurally similar to curcumin and also reduces levels of Bcl‐X_L_ in senescent cells.

Notably, extracts from two other plants—*Solidago virgaurea* subsp. *alpestris* and *Isatis tinctoria*, possess senolytic activity (Kim et al., 2022; Lammermann et al., 2018). However, their specific bioactive compounds have not been identified. *S. virgaurea* was selected as a candidate species based on anti‐inflammatory properties and investigated as a treatment for preventing age‐related skin dysfunction. While improved tissue function was largely attributed to the reduction in SASP expression, senolysis was also observed following chronic treatment (39 days) or at high concentrations. Later, the senolytic activity of *I. tinctoria* was identified from a screen of six plant extracts, which were also selected due to known anti‐inflammatory activity.

### 3.4 Heat‐shock protein inhibitors

Heat shock proteins (HSPs) play key roles in promoting correct protein folding, assembly, and function. Expression of HSPs rapidly increases in response to stress conditions, driving cell survival in‐part by heightened levels of anti‐apoptotic factors such as Bcl‐2 (Dubrez et al., 2020; Yenari et al., 2005). A screen of 97 autophagy modulators identified 17‐DMAG and seven other HSP90 inhibitors as senolytics, suggesting HSP90 as a key senolytic target. Treatment with 17‐DMAG was found to induce apoptosis in‐part by downregulating the anti‐apoptotic PI3K/AKT pathway (Fuhrmann‐Stroissnigg et al., 2017).

Another heat shock protein, CRYAB (α‐crystallin β chain), was discovered as a senolytic target through an extensive single‐cell RNA sequencing effort that looked at transcripts downregulated specifically in ABT‐263 treated senescent cells (Limbad et al., 2022). The known CRYAB inhibitor 25‐hydroxycholesterol (25HC) was then tested and confirmed to induce senolysis. 25HC demonstrated broad‐spectrum senolytic activity in mouse dermal cells and primary human cells from the lung, heart, liver, kidney, and articular cartilage.

### 3.5 BET family protein inhibitors

To date, the most extensive senolytic drug screening effort was conducted using the TAKEDA drug discovery library of 47,000 molecules (Wakita et al., 2020). The screen revealed four bromodomain and extra‐terminal domain family protein degraders (BETd) among the top hits. BET proteins are chromatin remodelers, influencing the transcription of various genes including cell cycle regulators and inflammatory factors (Kulikowski et al., 2021). Additional analysis identified the BETd ARV825 as a potent senolytic. The compound induced apoptosis in senescent cells by impairing non‐homologous end‐joining repair and increasing autophagic gene expression, which enhanced the incidence of DNA double‐stranded breaks. A separate study, which investigated the BET family inhibitor JQ1 alone, noted the role of the programmed cell death pathway ferroptosis in inducing senolysis (Go et al., 2021).

### 3.6 P53 stabilizers

P53 plays a key role in the DNA damage response and cell cycle regulation and can consequently activate apoptosis via multiple pathways (Fridman & Lowe, 2003). Although p53 is upregulated in pre‐senescent cells exposed to stress stimuli, after the transition to senescence, some cell types express p53 at reduced or even below basal levels (He, Li, et al., 2020). Several compounds that stabilize the protein possess senolytic activity. The inhibitor of human homolog of mouse double minute 2 (MDM2) RG‐7112 and ubiquitin‐specific protease 7 (USP7) inhibitors P5091 and P22077 are three senolytic compounds that reduce the degradation of p53 (Cherif et al., 2020; He, Li, et al., 2020).

### 3.7 Other repurposed anti‐cancer drugs

Although cellular senescence is distinctly different from the uncontrolled proliferation observed in cancer, both senescent and cancer cells share many common features. These include altered metabolism, resistance to apoptosis, and increased autophagy (Fouad & Aanei, 2017). Multiple anti‐cancer drugs have proven senolytic activity. GL‐V9, a synthetic flavonoid with anti‐tumor activity, was initially investigated under the hypothesis that it may induce senescence in breast cancer cells. However, it instead showed an unexpected senolytic activity (Yang, Tian, et al., 2021). GL‐V9 was subsequently found to induce apoptosis in multiple chemotherapy‐induced senescent cancer cell lines by increasing ROS and the number of mitochondria. Another senolytic, Panobinostat, is a histone deacetylase inhibitor developed for the treatment of multiple myeloma (Moore, 2016). The drug has since been found to induce cell death in various lung and head and neck cancer cells induced to senescence with chemotherapeutic agents (Samaraweera et al., 2017). Mitochondria‐targeted tamoxifen (MitoTam) is a chemotherapeutic and inhibitor of complex I of the electron transport chain that induces senolysis in multiple cell types. These include breast cancer cells, human retinal pigment epithelial cells, lung fibroblasts, and foreskin fibroblasts induced to senescence using various methods (Hubackova et al., 2019). MitoTam has a complex mechanism requiring low expression of adenine nucleotide translocase‐2 (ANT2), a protein that promotes mitochondrial integrity and biogenesis.

### 3.8 Cardiac steroids

The senolytic activity of cardiac steroids was identified by two separate studies that used high‐throughput screening to detect active compounds from commercial drug libraries (Guerrero et al., 2019; Triana‐Martinez et al., 2019). Pharmacologically active drug libraries contain molecules with a range of bioactivities and known pharmacokinetic properties, whereas collections of natural products offer molecules with evolved interactions in a biological system and can have a high molecular diversity (Shang et al., 2017). In total, 3742 substances from two drug libraries (Prestwick and LOPAC), two natural product libraries (GPNCL and SCREEN‐WELL®) and one library of venoms and venom‐derived peptides were tested. Multiple cardiac glycosides including bufalin, cinobufagin, convallatoxin, digitoxin, ouabain, peruvocide, and procillaridin A showed senolytic activity. Three toad extracts containing bufadienolide also showed senolytic properties. Other cardiac steroids without a glycoside chain have been shown to be senolytic, indicating that the feature is not obligatory. Confirmation of Na^+^ K^+^ ATPase as a senolytic target and the induction of pro‐apoptotic factor NOXA by cardiac glycosides was also reported. Yet, later studies revealed that cardiac glycosides exhibit minimal senolytic activity in cells with increased resistance to apoptosis (Deryabin et al., 2021).

### 3.9 PPARα agonists

The novel bioactivity of fenofibrate (a peroxisome proliferator‐activated receptor α agonist) was uncovered via a unique screening approach focused on therapeutics for cartilage degeneration and osteoarthritis (Nogueira‐Recalde et al., 2019). Approved drugs from the Prestwick chemical library were screened in senescent human chondrocytes for two separate outcomes: a reduction in expression of the senescent cell marker senescence‐associated β‐galactosidase (SA‐β‐gal) and an increase in autophagic flux. The latter is functionally informative as turnover at the lysosome is often defective in a degenerated cartilage. Fenofibrate was found to exhibit both properties.

### 3.10 Antibiotics

Azithromycin and roxithromycin are antibiotics that have shown some senolytic activity in human cells. This was revealed in a screen of 15 approved antibiotics in a model featuring BrdU‐treated MRC‐5 and BJ fibroblasts (Ozsvari et al., 2018). Moreover, the antibiotic nigericin was found to induce senolysis in several cell types (Deryabin et al., 2022). This is consistent with prior reports indicating it could disrupt various homeostatic systems in mammalian cells. The application of these antibiotics for senolytic therapy may be challenging due to the risks of accelerating antimicrobial resistance. However, these compounds may provide foundational insights into novel senolytic mechanisms and targets, allowing the development of analogs that retain senolytic properties but do not possess antibiotic activity.

## 4 METHODS FOR SENESCENCE INDUCTION IN CULTURED CELLS

The earliest report of senescent cells described this effect in serially passaged fibroblasts (Hayflick & Moorhead, 1961). This method for inducing senescence, termed replication‐induced senescence (RIS), has since been accepted as a common feature of somatic cells that contributes to aging (Campisi, 1997) and is largely caused by the shortening and destabilization of telomeres (Bodnar et al., 1998). Cells induced to senescence by replication are frequently used to screen or study senolytic compounds (Table 1). While the process of RIS is simple and requires no specialized equipment, many commonly used fibroblast cell lines (e.g. WI‐38 and IMR‐90 cells) require ~50 population doublings before becoming senescent (2022a, 2022b).

**TABLE 1:** Libraries screened for senolytic activity and identified classes of senolytics.

Senolytic class	Active compound(s)	Compound library tested to identify senolytic(s)	Cell model(s) used to identify activity	Ref.
Kinase inhibitors	Dasatinib	46 manually selected compounds known to target anti‐apoptotic pathways	HUVECs and preadipocytes induced by ionizing radiation	Zhu et al. (2015)
	Nintedanib, R406	Selleck Chem Kinase Inhibitor Library L1200 (*n* = 355)	HDFs induced by replicative exhaustion	Cho et al. (2020)
	Terreic acid^a^, PD‐98059^a^, daidzein^a^, Y‐27632^a^	SCREEN‐WELL® Kinase Inhibitor Library (*n* = 80)	HUVECs induced by oxidative stress, camptothecin treatment, and replicative exhaustion	Kusumoto et al. (2021)
Naturally occurring polyphenols	Quercetin	46 manually selected compounds known to target anti‐apoptotic pathways	HUVECs and preadipocytes induced by ionizing radiation	Zhu et al. (2015)
	Piperlongumine and analogs BRD4809, PL‐DI, PL‐7 and PL‐FPh	Manually selected compounds with bioactivities related to senescent cell viability	WI‐38 fibroblasts induced by ionizing radiation	Wang et al. (2016)
	Fisetin	Candidate study/10 flavonoid polyphenols	HUVECs induced by ionizing radiation, *Ercc1* ^*−/−*^ MEFs induced by oxidative stress, IMR‐90 fibroblasts induced by etoposide treatment	Yousefzadeh et al. (2018); Zhu et al. (2017)
	Curcumin	10 flavonoid polyphenols	*Ercc1* ^*−/−*^ MEFs induced by oxidative stress	Yousefzadeh et al. (2018)
	Curcumin analogs EF24, HO‐3867 and 2‐HBA	Candidate study also tested curcumin analog DIMC	WI‐38 fibroblasts induced by ionizing radiation	Li et al. (2019)
	o‐vanillin	Candidate study	naturally occurring senescent degenerated nucleus pulposus cells	Cherif et al. (2019)
	Procyanidin C1	46 natural compounds and plant extracts with putative anti‐aging properties	PSC27 prostate stromal cells induced by bleomycin treatment, replicative exhaustion, and oncogenic H‐RAS expression	Xu et al. (2021)
	Gingerenone A	4 plant extracts with anti‐inflammatory properties: Cat's claw, Devil's claw, Canadian goldenrod, and ginger	WI‐38 fibroblasts induced by ionizing radiation	Moaddel et al. (2022)
	*Solidago virgaurea* subsp. *alpestris*	Candidate study	HDFs induced by oxidative stress (senolysis observed after a chronic treatment period of 39 days)	Lammermann et al. (2018)
	*Isatis tinctoria* extract (active components not analyzed)	Six plant extracts with anti‐inflammatory properties: *Isatis tinctoria, Rosmarinus officinalis, Rosa, Prunus persica, Nelumbo nucifera, Citrus unshiu Marcov*	HDFs induced by replicative exhaustion	Kim et al. (2022)
BCL‐2 family inhibitors	ABT‐263	37 manually selected compounds with bioactivities related to senescent cell viability	WI‐38 fibroblasts induced by ionizing radiation	Chang et al. (2016)
	A1331852, A1155463	Candidate study	HUVECs and IMR‐90 fibroblasts induced by ionizing radiation	Zhu et al. (2017)
	ABT‐737	Candidate study	IMR‐90 fibroblasts induced by exhaustive replication, etoposide treatment and oncogenic H‐Ras expression	Yosef et al. (2016)
	S63845, UM177, AZD5991	Candidate study	PC3 and LNCaP prostate tumor cells induced by treatment with docetaxel and palbociclib	Troiani et al. (2022)
Heat shock protein inhibitors	17‐DMAG and other HSP90 inhibitors	SCREEN‐WELL® autophagy library (*n* = 97)	*Ercc1* ^*−/−*^ MEFs induced by oxidative stress	Fuhrmann‐Stroissnigg et al. (2017)
	25‐hydroxycholesterol	Candidate study, also tested NCI‐41356	Fibro‐adipogenic progenitors, satellite cells, and skeletal muscle myoblasts induced by doxorubicin treatment	Limbad et al. (2022)
BET family protein degraders	ARV825, I‐BET151, I‐BET762, JQ1, OTX015, PFI‐1	TAKEDA drug discovery library (*n* = 47,000)	IMR‐90 fibroblasts induced by oncogenic H‐Ras expression	Wakita et al. (2020)
	JQ1	Candidate study	HDFs induced via bleomycin treatment	Go et al. (2021)
P53 stabilizers	RG‐7112	Candidate study	Naturally occurring senescent annulus fibrosus and nucleus pulposus cells	Cherif et al. (2020)
	USP7 inhibitors P5091 and P22077	Candidate study	WI‐38 fibroblasts induced by ionizing radiation and exhaustive replication	He, Li, et al. (2020)
Other repurposed anti‐cancer drugs	GL‐V9	Candidate study	Naturally occurring senescent MDA‐MB‐231 breast cancer cells and MEFs induced by exhaustive replication	Yang, Tian, et al. (2021)
	Panobinostat	Candidate study	A549 lung carcinoma cells induced by paclitaxel and cisplatin treatment	Samaraweera et al. (2017)
	MitoTam	Candidate study	4 T1 and MCF7 breast cancer cells induced by doxorubicin treatment, RPE‐1 and HFP‐1 cells induced by BrdU treatment, and BJ fibroblasts induced by exhaustive replication	Hubackova et al. (2019)
Cardiac steroids	Bufalin, cinobufagin, convallatoxin, digoxin, digitoxin, ouabain, peruvocide, proscillaridin A	LOPAC and Prestwick libraries of pharmacologically active compounds, GreenPharma and SCREEN‐WELL® natural compound libraries, and one library of venoms and venom‐derived peptides (*n* = 3742)	IMR‐90 fibroblasts induced by oncogenic H‐Ras expression and etoposide treatment, A549 lung adenocarcinoma cells induced by bleomycin treatment, and SK‐MEL‐103 melanoma cells induced by palbociclib treatment	Guerrero et al. (2019); Triana‐Martinez et al. (2019)
PPRα agonists	Fenofibrate	Prestwick library of pharmacologically active approved compounds (*n* = 1120)	T/C‐28a2 chondrocytes induced by treatment with IL‐6	Nogueira‐Recalde et al. (2019)
Antibiotics	Azithromycin, roxithromycin	15 manually selected approved drugs	MRC‐5 and BJ fibroblasts induced by treatment with BrdU	Ozsvari et al. (2018)
	Nigericin	Candidate study	Endometrial and Warton jelly‐derived MSCs induced by oxidative stress, endometrial MSCs induced by exhaustive replication, dental pulp MSCs induced by doxorubicin treatment, A459 lung adenocarcinoma cells induced by etoposide treatment	Deryabin et al. (2022)

Abbreviations: HDF, human dermal fibroblast; HUVEC, human umbilical vein endothelial cell; MEF, murine embryonic fibroblast; MSC, mesenchymal stem cell.

^a^ Senotherapeutic activity identified but not tested specifically for senolytic activity.

**FIGURE 2:**
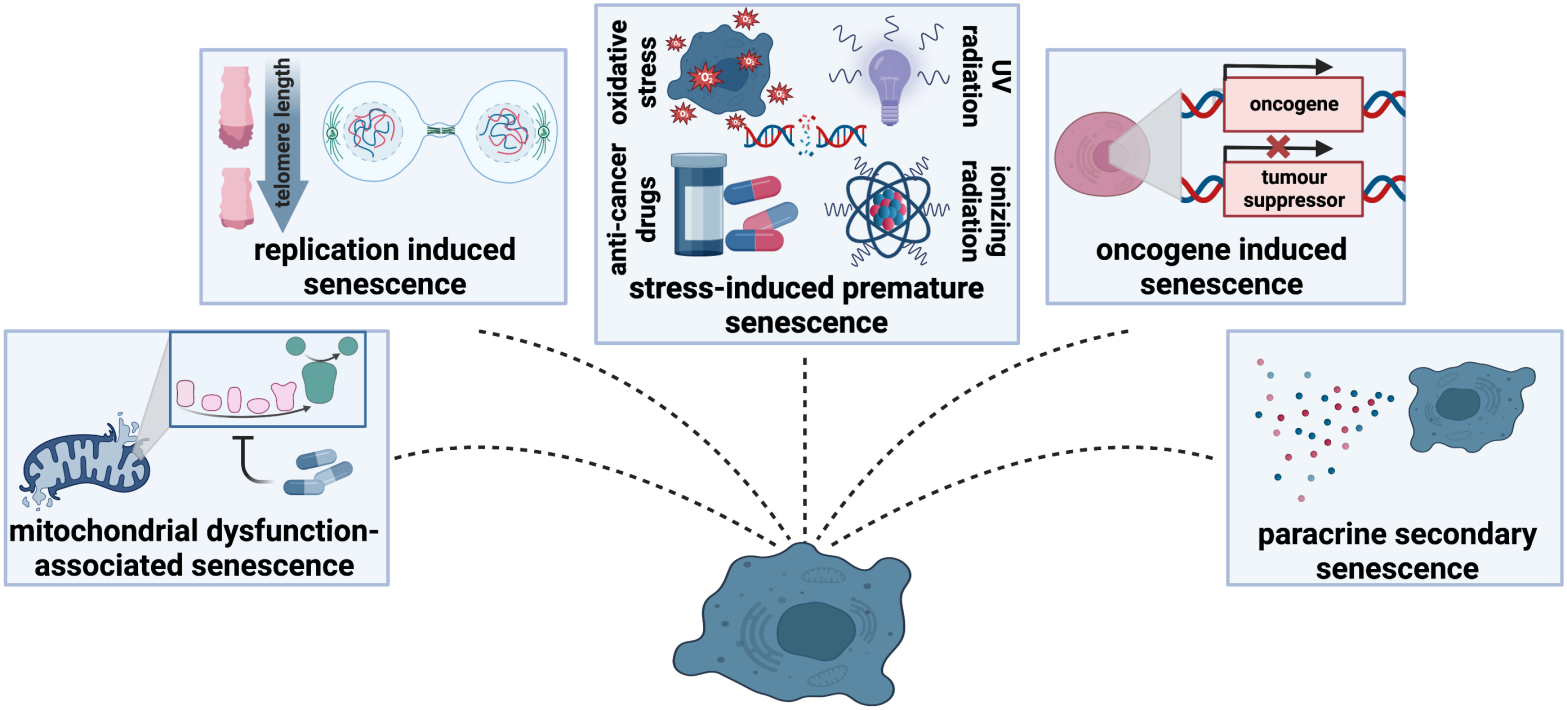
Methods for inducing a senescent phenotype.

In contrast, stress‐induced senescence (SIS) can be achieved through exposure to DNA‐damaging agents such as oxidative stress (Wang et al., 2013), ionizing radiation (Noren Hooten & Evans, 2017), ultraviolet radiation (Martic et al., 2020) and anti‐cancer drugs (Neri et al., 2021) (Figure 2). While both RIS and SIS induce the DNA damage response, SIS causes DNA damage not only at telomeres but also randomly across the genome (de Magalhaes & Passos, 2018). Despite this, damage to telomeres is difficult to repair and is a driving factor of SIS regardless of telomere length and expression of the human telomerase enzyme (hTERT) (de Magalhaes & Passos, 2018). Some induction methods such as radiation or oxidative stress may reflect age‐related conditions caused by routine environmental exposures (Debacq‐Chainiaux et al., 2012; Liguori et al., 2018). In the context of cancer therapy, specific stress stimuli including ionizing radiation and anti‐cancer drugs are of particular interest. While these methods are used to treat cancers, senescent cells arise as a side‐effect and can cause treatment resistance and tumor recurrence (Yang, Liu, et al., 2021). Novel tumor therapies combining chemotherapeutics and senolytics are therefore of interest, with recent pre‐clinical studies reporting combination therapy to reduce tumor growth, metastases, and drug toxicity (Estepa‐Fernández et al., 2023; Galiana et al., 2020).

Oncogene‐induced senescence (OIS) is of similar relevance to cancer therapy. OIS occurs as a result of activated oncogenes such as *H‐ras*, *K‐ras*, and *B‐raf* or inactivated tumor suppressors such as *PTEN* and *NF1* (Courtois‐Cox et al., 2008). Following a brief period of accelerated proliferation, multiple mechanisms cause cells to convert to a senescent state. These include the DNA damage response triggered by accelerated replication and increased ROS, ROS‐induced p38/PRAK signaling, formation of senescence‐associated heterochromatin foci (SAHF), and negative feedback signaling, which suppresses growth pathways (Courtois‐Cox et al., 2008). In vitro, ectopic expression of the constitutively active H‐RasV12 protein via plasmid transfection is the most common model of OIS (Ogrunc et al., 2014).

Although less common, secondary senescence can occur via paracrine exposure to specific SASP factors. This reflects changes that occur in tissues during aging and thus may be used to study senolytic activity in specific biological environments. For example, the assay system employed to identify senolytics for osteoarthritis treatment used the pro‐inflammatory SASP factor IL‐6 to induce senescence in chondrocytes (Nogueira‐Recalde et al., 2019). Elevated IL‐6 levels in the cartilage of osteoarthritis patients contribute to disease development (Wiegertjes et al., 2020) and IL‐6 exposure in chondrocytes induces a distinct response involving decreased autophagic flux and activated mTOR signaling (Nogueira‐Recalde et al., 2019).

Induction of dysfunctional mitochondria is another biologically relevant, yet uncommon method for generating senescent cells. During aging, defective mitochondria accumulate in the heart, skeletal muscle, colon, and neurons (Trifunovic & Larsson, 2008) and are associated with certain chronic diseases such as cardiovascular disease, type 2 diabetes, and cancer (Diaz‐Vegas et al., 2020). In cultured cells, mitochondrial dysfunction‐associated senescence (MiDAS) can be induced by electron transport chain inhibitors, knockdown of mitochondrial chaperone HSPA9, or depletion of mitochondrial DNA. Notably, senescent cells induced by these stimuli activate pathways and possess a SASP clearly distinct from RIS, SIS, and OIS (Wiley et al., 2016).

## 5 CELL LINES USED TO STUDY SENOLYTICS

Currently, the most common cells used in senolytic screens are the fetal lung fibroblast cell strains WI‐38 and IMR‐90 (Table 2). These cells offer advantages such as purchasability, ease of culturing, consistent genetic background, no disease state, and a finite lifespan, allowing replicative senescence to be induced by serial passage (Li et al., 2019). WI‐38 cells are extensively characterized, being used to study senescence since their derivation by L. Hayflick in 1962 (Hayflick, 1965, 1984). Alternatively, *Ercc1* ^*−/−*^ MEFs are often used as a murine‐derived fibroblast cell model and as a precursor for testing senolytics in *Ercc1* ^*−/Δ*^ progeroid mice (Weeda et al., 1997). These cells are deficient in the ERCC1‐XPF DNA repair complex, resulting in increased sensitivity to DNA‐damaging agents. Consequently, induction of senescence is rapid and commonly performed by exposure to 20% O_2_ for five passages (Fuhrmann‐Stroissnigg et al., 2017; Yousefzadeh et al., 2018).

**TABLE 2:** Conventional cell lines and primary cells used to screen for senolytic activity.

Cell name	Morphology	Tissue of origin	Reasons for use	Screening examples
WI‐38	Fibroblast	Human lung	First cell line used to study replicative senescence, well‐characterized senescence phenotype and established senescence induction methods	Chang et al. (2016); He, Li, et al. (2020); Li et al. (2019); Moaddel et al. (2022); Wang et al. (2016)
IMR‐90	Fibroblast	Human lung	Similar properties to WI‐38 and commonly used as an alternative cell line	Guerrero et al. (2019); Wakita et al. (2020); Yosef et al. (2016); Zhu et al. (2017)
Human umbilical vein endothelial cell (HUVEC)	Endothelial	Vein of human umbilical cord	Well‐established senescence induction methods, cells senesce quickly following serial passage, established model for aging endothelial cells and vasculature	Kusumoto et al. (2021); Zhu et al. (2017); Zhu et al. (2015)
Human dermal fibroblasts (HDFs)	Fibroblast	Human skin	Well‐established senescence induction methods, used as a model for studying skin tissue dysfunction	Cho et al. (2020); Go et al. (2021); Kim et al. (2022); Lammermann et al. (2018)
BJ fibroblasts	Fibroblast	Human foreskin	Well‐established senescence induction methods, used as a cellular model of skin aging	Hubackova et al. (2019); Ozsvari et al. (2018)
*Ercc1* ^*−/−*^ mouse embryonic fibroblasts	Fibroblast	Embryos of pregnant *Ercc* ^*−/* Δ^ mice	Mutation in ERCC1 DNA repair protein is associated with a rapid aging phenotype, cells senesce quickly following exposure to atmospheric O_2_, used as an in vitro precursor to senolytic trials in the *Ercc* ^*−/ Δ*^ mouse model of human progeroid syndrome	Fuhrmann‐Stroissnigg et al. (2017); Yousefzadeh et al. (2018)
Preadipocytes	Fibroblast	Human adipose	Senescent preadipocytes are abundant in human tissue and contribute to impaired adipogenesis and age‐related metabolic dysfunction	Zhu et al. (2015)
Annulus fibrosus and nucleus pulposus cells	Fibroblast	Human intervertebral discs	Senescent cells from intervertebral discs contribute to inflammation and back pain	Cherif et al. (2019); Cherif et al. (2020)
Various cancer cell lines	Various	Various (breast, lung, brain, liver, mouth, pharynx)	Senescent cells are a feature of tumors and contribute to cancer progression and treatment resistance, common anti‐cancer therapies induce cellular senescence	Hubackova et al. (2019); Samaraweera et al. (2017); Triana‐Martinez et al. (2019); Troiani et al. (2022); Yang, Tian, et al. (2021)

Other cell strains have been used for their relevance to conditions caused or exacerbated by senescent cells. The first senolytics D and Q were initially tested in primary preadipocytes due to their abundance in humans and the significance of fat tissue in disease (Zhu et al., 2015). Another strain of primary cells, HUVECs, were also included and have been previously studied as a model for vascular aging (Wagner et al., 2001). Other studies have focused on the contribution of senescent cells to skin aging, dysfunction, and tumor formation and have used primary HDFs and keratinocytes fibroblasts as relevant cell types (Kim et al., 2022; Lammermann et al., 2018).

Multiple publications have focused on specific chronic diseases with major healthcare burdens. One looked at senolytic treatments for osteoarthritis and used an immortalized chondrocyte cell line (TC28a2) due to the involvement of senescent chondrocytes in cartilage degeneration. Studies focusing on intervertebral disc (IVD) degeneration, have used annulus fibrosus and nucleus pulposus cells isolated from human IVDs (Cherif et al., 2019; Cherif et al., 2020). A multitude of cancer cell lines has also been employed due to the role of senescence in tumor growth and resistance to chemotherapeutics (Hubackova et al., 2019; Park et al., 2021; Samaraweera et al., 2017; Triana‐Martinez et al., 2019; Yang, Tian, et al., 2021).

## 6 SENOLYTIC ACTIVITY CAN BE CELL‐TYPE AND ASSAY‐DEPENDENT

The capacity of cell assays to respond to a particular senolytic is dependent on both the cell line and the method used for senescence induction. The initial identification of a senolytic is frequently performed with WI‐38 or IMR‐90 fibroblasts or a cell type related to a pathogenic condition. Subsequently, the capacity of a senolytic to have a broad activity requires confirmation in a diverse range of cell types. Such an approach was used to assess the senolytic spectrum of 25HC, where the metabolite was tested in nine different cell types from mouse and human origin, including cells from skeletal muscle, lung, heart, liver, kidney, and articular cartilage tissue (Limbad et al., 2022).

Cell type can have a considerable impact on the utility of a senolytic drug. For example, the compound JQ1 was initially identified in a high‐throughput screen in oncogene‐induced senescent IMR‐90 fibroblasts (Wakita et al., 2020) and later studied in bleomycin‐treated HDFs (Go et al., 2021). However, a prior study had already screened JQ1 and found no associated senolytic activity in irradiated WI‐38 fibroblasts (Chang et al., 2016). Moreover, the Prestwick chemical library was screened in two separate studies, with two distinct outcomes. One identified cardiac glycosides to be active in lung cancer cells (Triana‐Martinez et al., 2019) and the other reported the activity of fenofibrate in chondrocytes (Nogueira‐Recalde et al., 2019). Senotherapeutics resveratrol and curcumin have been reported to paradoxically *induce* senescence in certain cell types (Maria & Ingrid, 2017). Thus, the general applicability of new senolytic compounds should ideally be validated in multiple in vitro and in vivo systems.

The type of stress stimuli used also influences senolytic activity, although this appears to have less of an impact compared to cell type. For example, PL is a more potent senolytic in replication‐induced WI‐38 cells compared to irradiated or oncogenic Ras‐induced WI‐38 cells (Wang et al., 2016). Whereas for ABT‐263, potency is higher in irradiated and Ras‐induced senescent WI‐38 cells compared to those induced by replication (Chang et al., 2016). Differences in senolytic activity are reflected in gene expression profiles. A study analyzing transcriptome profiles of six fibroblast strains and multiple senescence induction stimuli found variation was more strongly correlated with tissue type compared to the induction method (Hernandez‐Segura et al., 2017). Transcriptomes were also temporally dynamic, with cellular responses changing at varying intervals after senescence induction.

## 7 ASSAYS TO CONFIRM INDUCTION OF A SENESCENT PHENOTYPE

A critical feature of high‐quality senolytic screens is the efficient generation of experimentally validated and quantified senescent cells. The SA‐β‐gal assay is the most common method for detecting senescent cells. β‐galactosidase is a lysosomal enzyme active in proliferating cells and has an optimal pH between 4.0 and 4.5. Senescent cells, however, exhibit an increased expression of β‐gal and this high level of enzyme activity can be detected at an above‐optimal pH, termed SA‐β‐gal activity (Lee et al., 2006). The assay is typically performed at pH 6.0, however, the ideal pH differs between cell types. Various substrates for β‐gal can be used, with the most common being chromogenic substrate 5‐bromo‐4‐chloro‐3‐indoyl β‐D‐galactopyranoside (X‐gal). X‐gal assays are simple and fast to set up, though quantification is generally time‐consuming and performed by manually counting cells under a light microscope (Debacq‐Chainiaux et al., 2009). In some cases, digital image analysis can streamline the process (Shlush & Selig, 2013). Conversely, fluorogenic substrates such as 5‐dodecanoylamino‐fluorescein di‐β‐D‐galactopyranoside (C_12_FDG) or 4‐methylumbelliferyl‐β‐d‐galactopyranoside (MUG) can be detected by flow cytometry or fluorescence microscopy. Although the protocols can be more time‐consuming and/or complex to set‐up, quantification is automated (Debacq‐Chainiaux et al., 2009). A chemiluminescent method using the substrate Galacton has also been reported, with enzymatic activity of cell lysates measured using a luminometer and normalized to protein content (Bassaneze et al., 2008).

**FIGURE 3:**
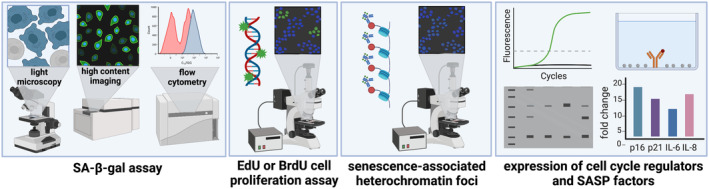
Methods for confirming induction of a senescent phenotype.

Despite the widespread use of SA‐β‐gal as a senescence biomarker, issues regarding its lack of specificity have been raised. For example, excess β‐gal expression can be detected in quiescent cells induced by serum starvation or contact inhibition (Cho & Hwang, 2012; Yang & Hu, 2005). Moreover, some non‐senescent cell types such as osteoclasts and immature neurons are also positive for SA‐β‐gal (Kopp et al., 2007; Piechota et al., 2016). To strengthen confidence in senescent cell identification, multiple biomarkers have been used. These include gene and protein expression of the cell cycle regulators p16^INK4a^ (p16) and p21^CIP1^ (p21) and expression of SASP factors relevant to the cell line and induction method. Increased prevalence of senescence‐associated heterochromatin foci markers such as H3K9Me2 and H3K9Me3 and the DNA damage foci marker γH2AX can also be used as senescence indicators. Moreover, the absence of proliferation can be shown by the lack of incorporation of fluorescent nucleotides such as 5‐ethynyl‐2′‐deoxyuridine (EdU) or antibody labeled 5‐bromo‐2‐deoxyuridine (BrdU). Detailed discussions of known senescence‐associated biomarkers and methods for detection can be found elsewhere (Gonzalez‐Gualda et al., 2021; Noren Hooten & Evans, 2017). However, like SA‐β‐gal activity, these indicators may only be expressed in specific cell types and may also be present in non‐senescent cells under certain conditions. Figure 3 summarizes the four main approaches for detecting senescent cells (SA‐β‐gal assay, proliferation assay, detection of SAHF, and expression analysis of cell cycle regulators and SASP factors).

As there are no universal markers of senescence, when testing senolytics it is critical to include appropriate controls to minimize false positives and negatives. These include non‐senescent controls in the form of proliferating low‐passage cells and quiescent cells that have not been exposed to the relevant senescence‐inducing stimulus. Vehicle controls for both proliferating and senescent populations are also required. Previously verified senolytics should be included as positive controls to validate the assay system and can be used to generate threshold values to select lead compounds. As senolytic activity varies according to cell type and senescence‐inducing stimulus, it is beneficial for initial studies to use cell models with known responses before trialing non‐standard cell types. Performing a cell viability measurement at day 0 of drug treatment can also be incorporated into screening protocols to improve the accuracy of results, allowing post‐treatment data of individual wells to be normalized to pre‐treatment data (Zhu et al., 2015). The calculation not only accounts for variability between wells, but also confirms the absence of proliferation in senescent populations.

## 8 EMERGING TECHNOLOGIES APPLICABLE TO SENOLYTIC SCREENING

### 8.1 Principles of senolytic screens

**FIGURE 4:**
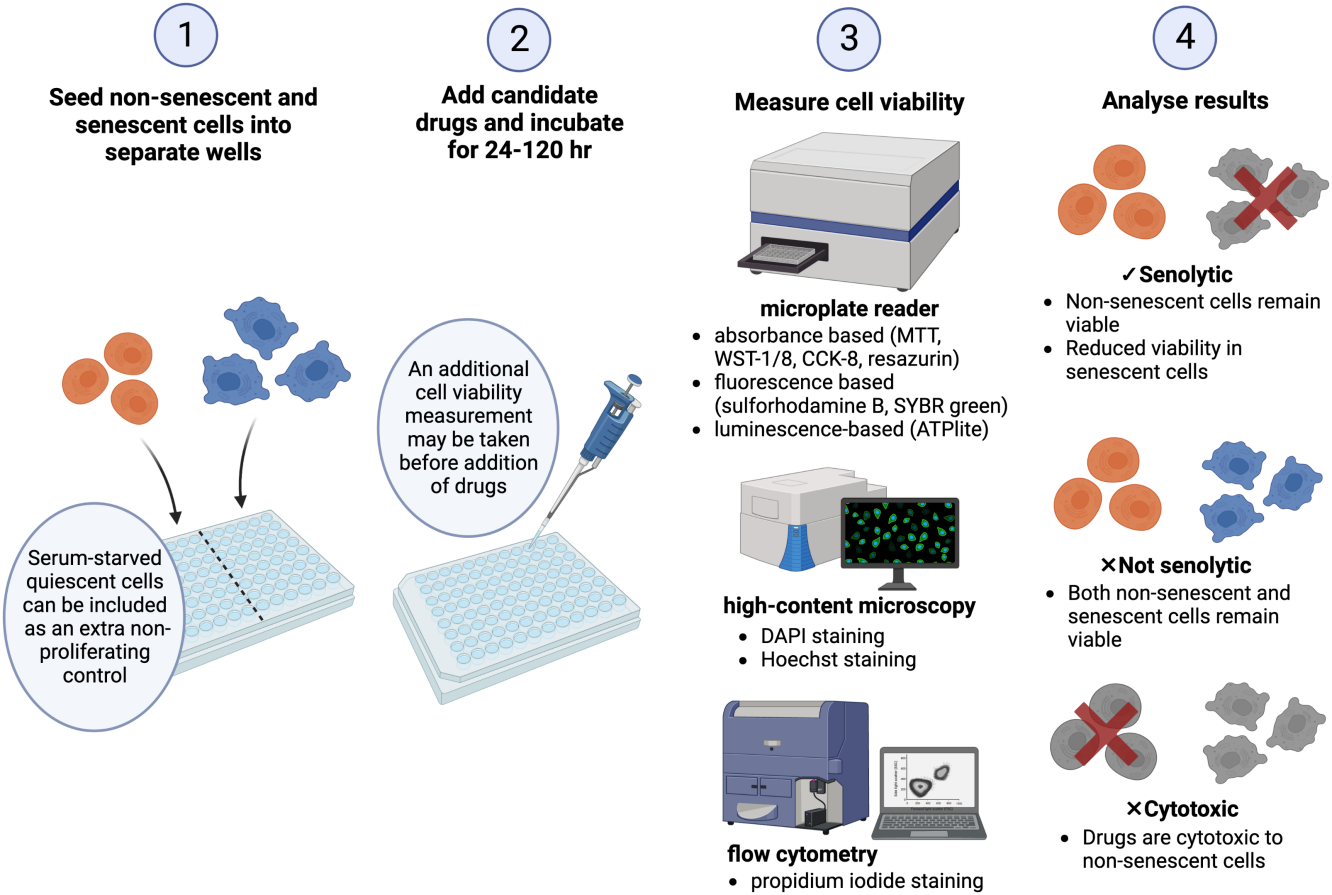
The basic workflow for detecting novel senolytic compounds.

Existing workflows compare the impact of candidate senolytics on non‐senescent vs senescent cells. Figure 4 illustrates the four main steps: (1) seeding non‐senescent and senescent cells into wells, (2) incubating cells with candidate drugs, (3) measuring cell viability, and (4) analyzing results. Lead compounds should reduce the viability of senescent cells while exhibiting minimal cytotoxicity in non‐senescent cells. Viability is typically measured indirectly using an enzymatic reaction based on cellular metabolism or ATP levels, or dyes that bind protein or DNA content. Absorbance, fluorescence, or luminescence of the reaction product is then detected using a plate reader. Advanced approaches have recently emerged with a greater accuracy, efficiency, and capacity to screen large compound libraries.

### 8.2 Single‐cell screening methods

Single‐cell analysis encompasses a suite of potent techniques for directly quantifying the total cell number and the percentage of senescent cells simultaneously. Methods for single‐cell quantification include flow cytometry compatible dye exclusion and high‐content fluorescent microscopy of stained cells counted by automated image capturing and analysis. While single‐cell techniques require specialized equipment and are typically more costly than population‐based methods, testing of large compound libraries is more efficient and accurate. To simultaneously quantify senescent cells, SA‐β‐gal activity can be detected using the fluorogenic substrate C_12_FDG (Fuhrmann‐Stroissnigg et al., 2017; Nogueira‐Recalde et al., 2019; Yousefzadeh et al., 2018). Consequently, senolytic compounds can be differentiated from those which reduce the senescent phenotype without eliminating cells (senomorphic). Senolytic compounds are identified as those which reduce the number of senescent cells and while maintaining the number of non‐senescent cells, whereas senomorphic compounds maintain total cell numbers while reducing SA‐β‐gal positive cells. Additional markers can also be included to detect senolytics that influence specific pathways of interest. For example, one senolytic screen used high‐content fluorescent microscopy to detect increased levels of microtubule‐associated protein 1A/1B‐light chain 3 (LC3). LC3 is a marker of autophagic flux (Nogueira‐Recalde et al., 2019), a process defective in age‐related disease (Dodig et al., 2019). It was hypothesized that senolytic compounds which simultaneously increase autophagic flux may provide enhanced therapeutic benefits.

A further development in single‐cell methods is protocols that co‐culture senescent and non‐senescent control cells in the same well, mitigating biases caused by separating these populations. This approach eliminates potential distortions introduced by the active cell cycle of proliferating control cells or low‐serum conditions required for quiescent control cells. Quantification can be performed by detecting SA‐β‐gal in single cells (Fuhrmann‐Stroissnigg et al., 2019) or by co‐culturing populations of non‐senescent and senescent cells transduced with distinct fluorescent protein reporters such as GFP and RFP (Triana‐Martinez et al., 2019). As protein reporters do not rely on dyes or substrates to quantify cells, changes in viability overtime can be measured simply and rapidly.

### 8.3 Big data and high‐performance computing in senolytic discovery

Advancements in computational power and memory have allowed for further insight into senescent cell biology and potential senolytic targets. Limbad et al. leveraged single‐cell RNA‐sequencing technology to identify two novel senolytic targets CRYAB and HMOX1. The targets were identified by detecting transcripts upregulated in senescent fibro‐adipogenic progenitors and satellite cells, that were consequently downregulated upon senolysis with ABT‐263 (Limbad et al., 2022). Consequently, the known CRYAB inhibitor 25HC was tested and validated as a senolytic drug. While this study demonstrates an advanced senolytic discovery method, its success was limited by a few known CRYAB and HMOX1 inhibitors. While HMOX1 was experimentally confirmed as a senolytic target, neither of the tested inhibitors was sufficiently senolytic to be practicable. In addition to RNA sequencing, other computationally intensive methods such as virtual screening can be used to predict potential senolytic candidates in silico. Such techniques require high‐quality experimental data regarding senolytic mechanisms, targets, or potency of active compounds. While currently limited in their scope, these approaches will be greatly enabled by future experimental research.

Computer processing power has also been harnessed to incorporate image‐based machine learning methods into high‐throughput senolytic drug screens. One study developed a morphology‐based senescence scoring system titled Deep‐SeSMo to quantify senescence at single‐cell resolution (Kusumoto et al., 2021). A convolutional neural network was trained with phase‐contrast microscopy images of HUVECs induced to senescence by replicative exhaustion, oxidative stress, or camptothecin. The model predicted senescence scores with high accuracy and rapid calculation times (0.08–0.1 ms/cell). DeepSeSMo continued to perform well when trained on images acquired from an external institute and when using an alternative fibroblast cell line. When used to conduct a screen for senotherapeutic drugs, it successfully identified known and novel anti‐senescence compounds. Using morphology is both label‐free and enables both senescent and non‐senescent cells to be assayed in the same well. Nevertheless, algorithm training requires suitable input data, appropriate guidance, and operator expertise.

### 8.4 Emerging screening approaches

A recent study reported dihomo‐15d‐PGJ2 as the first marker of senolysis, unlocking the potential for novel screening methods that do not involve cell viability assays (Wiley et al., 2021). This bioactive lipid that is upregulated in senescent cells and reinforces cell cycle arrest and promotes SASP factor expression. Unlike traditional senescence markers, the study focused on measuring secreted lipid levels in culture media and plasma of mice in response to treatment with senolytics. While there is potential for dihomo‐15PGJ2 to be used in screening for senolytics (Limbad et al., 2022), there are currently no published studies using this approach. Dihomo‐15PGJ2 has only been confirmed as a biomarker in mitochondrial dysfunction‐induced IMR‐90 cells and in irradiated IMR‐90, HUVEC, and HEPG2 cells. Therefore, its use as an indicator of senolysis must be validated in the relevant cell models prior to screening.

## 9 INVESTIGATING SENOLYTICS' MECHANISMS OF ACTION

After screening for novel senolytics, additional assays are needed to interrogate their mechanism of action and confirm whether they affect conventional molecular targets. Viability assays that quantify individual cells can be combined with senescence biomarkers such as SA‐β‐gal or cell cycle arrest markers such as p16, p53, and p21. This is necessary to show cell death is specific to senescent cells. Given there are no universal senescence‐specific biomarkers, simultaneous monitoring of multiple factors is best practice. Additionally, gene or protein expression of SASP factors in response to senolytic treatment can be measured using standard techniques such as qPCR, western blot, enzyme‐linked immunosorbent assay, or immunostaining. As senolytics are specific to cell type and induction method, one may also consider testing activity spectrum using a range of cell types induced to senescence with various stimuli.

Apoptosis and ROS‐dependent death are recognized as key cell death pathways in senolysis. Apoptosis can be quantified in individual cells by annexin V staining or in pooled cells by measuring caspase expression using western blot, immunostaining, or with a caspase‐specific luminescent substrate such as DEVD‐aminoluciferin. Co‐incubation with a pan‐caspase inhibitor such as Q‐VD‐OPh is then required to confirm whether senolytic activity is specifically apoptosis‐dependent (Chang et al., 2016). ROS‐dependent cell death is another commonly investigated pathway. Cellular production of these reactive molecules plays a role in mediating various cell death types including apoptosis, ferroptosis, and necroptosis, however, production is not always essential for the activation of these pathways. To determine whether senolysis is ROS‐dependent, oxidation of a fluorogenic substrate can be used to compare ROS levels in proliferating vs senescent cells treated with the senolytic of interest. To confirm ROS‐dependence, co‐incubation with the senolytic compound and a neutralizing free‐radical scavenger is performed. This method was used to confirm the ROS‐dependent senolytic activity of procyanidin C1 (Xu et al., 2021). ROS induction increased in treated senescent cells and cell death was suppressed with free‐radical scavenger HS‐1793. A similar experiment was conducted with PL and conversely found senolysis was ROS‐independent. Although PL treatment induced ROS in senescent cells, co‐treatment with the antioxidant γ‐tocotrienol did not prevent cell death (Wang et al., 2016). Although infrequently reported, recent studies have shed light on the involvement of ferroptosis (Go et al., 2021) and pyroptosis (Deryabin et al., 2022) in senolysis, suggesting the significance of exploring alternative pathways.

Although no standardized methods exist for specifically studying senolytic mechanisms, large‐scale transcriptomic or proteomic analysis is usually performed to identify novel pathways and binding targets. To investigate modulated pathways, standard molecular biology techniques can be used, such as RNA sequencing for gene analysis or mass‐spectrometry for protein analysis. Comparisons are then made between untreated senescent cells and those treated with the senolytic of interest. As an example, differentially expressed genes in senescent cells treated with and without the senolytic ARV825 were revealed with RNA sequencing. Down‐regulation of DNA repair gene *xrcc4* was identified, consistent with the observed impairment to non‐homologous end joining (Wakita et al., 2020).

Further experimental approaches may be required to address unconventional binding targets. If there is a pre‐existing hypothesis, surface plasmon resonance or similar methods may confirm binding to the putative target. However, where there is no hypothesized target, more extensive unbiased binding studies are needed. Typically, this involves using the senolytic compound as a bait and analyzing bound proteins from the lysates of non‐senescent vs senescent cells. For example, Zhang et al. (2018) employed affinity chromatography to identify OXR1 as the senolytic target of PL. A pull‐down assay using a PL chemical probe was conducted, followed by mass‐spectrometry sequencing to compare bound proteins in non‐senescent and senescent fibroblasts. A follow‐up *OXR1* knockdown experiment was then used to confirm the senolytic target. There is also scope for in silico modeling methods such as docking and molecular dynamics to predict novel binding targets. However, in the context of senolytics, such computational approaches have not yet been reported.

Overall, the inherent heterogeneity of senescent cells complicates standardized workflows. Transcriptome and protein profiles vary across cell types, within the same cells in response to various senescence inducers, at different time points post‐induction, and even between experiments conducted in the same lab (Althubiti et al., 2014; Hernandez‐Segura et al., 2017). Upregulated factors are often shared with other cellular states, such as quiescence, adding complexity to identification of senescence‐specific biomarkers (Hernandez‐Segura et al., 2018). Consequently, senolytic activity varies significantly across cell types and experimental conditions. Thus, multiple assays are required to validate findings and the approach should be context and application specific.

## 10 CONCLUSION

Since the discovery of the first senolytics, both hypothesis‐driven studies and compound library screens have identified a range of novel senolytics which act via various mechanisms and target multiple cell types. Ultimately, these compounds still face hurdles in terms of medical translation. They must demonstrate effective targeting of deleterious senescent cells, appropriate bioavailability, and a favorable safety profile. Even repurposed drugs with known safety profiles require trials to optimize dosage regimens and assess efficacy in the context of senescence and associated chronic diseases. Currently, clinical trials are mainly limited to D + Q or fisetin with few progressing to phase III or beyond (Chaib et al., 2022).

Given that senescent cells are a common feature of many age‐related pathologies, senolytics have the potential to alleviate a multitude of diseases. However, due to varying efficacies to stress‐induced responses and in diverse cell types, applying a single senolytic drug may be ineffective at alleviating complex pathologies. Continued research into the effect of senolytics and senolytic cocktails in the context of specific conditions and exploring combination therapies will be needed to advance the field.

## AUTHOR CONTRIBUTIONS

Helen Power drafted all manuscript sections and generated figures. Aaron Schindeler formulated the structure and substantially revised the manuscript. Peter Valtchev and Fariba Dehghani reviewed and edited the manuscript. All authors have read and agreed to the published version of the manuscript

## FUNDING INFORMATION

The authors received no financial support for the authorship and/or publication of this article.

## CONFLICT OF INTEREST STATEMENT

The authors have no competing interests to declare.
